# Worsening effect of testosterone deficiency on males with heart failure with preserved ejection fraction

**DOI:** 10.1186/s12902-022-01249-3

**Published:** 2022-12-19

**Authors:** Ahmed Hamam, Mahmoud Abou-Omar, Hanem Rabah, Haidy Khattab, Ahmed Alaarag

**Affiliations:** 1 Department of internal medicine, Armed Forces College of Medicine, Cairo, Egypt; 2 Cardiovascular medicine department, Faculty of Medicine, Tanta University, Tanta, 31511 Egypt; 3 Faculty of Medicine, Department of medical biochemistry, Tanta University, Tanta, Egypt; 4 Faculty of Medicine Department of medical physiology, Tanta University, Tanta, Egypt

**Keywords:** HFpEF, Testosterone, NYHA class

## Abstract

**Background:**

Heart failure with preserved ejection fraction (HFpEF)is challenging. Patients usually have normal LV size and ejection fraction. This clinical syndrome develops from a complex interaction of several risk factors that cause organ dysfunction and clinical symptoms. There’s evidence that testosterone deficiency is associated with a worse cardiometabolic profile and increased inflammatory markers. We thought that these changes might have an impact on heart failure pathogenesis. We aimed to study the relationship between testosterone level and symptoms in HFpEF.

**Methods:**

We studied 120 male patients with HFpEF. According to New York Heart Association (NYHA), patients were classified into I, II and III classes; class IV patients were excluded. All patients were subjected to clinical and echocardiographic examinations. In addition, we measured serum testosterone, cardio-metabolic profile, intracellular adhesive molecule-1(ICAM-1), P-selectin and nitric oxide (NO) levels.

**Results:**

Patients with testosterone deficiency had worse NYHA class and higher BNP *P =* (0.001). Additionally, they had a significantly worse metabolic profile; higher total cholesterol, triglycerides, LDL cholesterol, fasting insulin and HOMA-IR *P =* (0.005, 0.001, 0.001, 0.001), respectively.

Also, they had higher inflammatory markers and worse endothelial functional parameters; (ICAM-1, NO and P- selectin) *P =* (0.001).

Age, BNP and testosterone deficiency can be used as independent predictors of NYHA class III symptoms with a Testosterone cutoff value of 2.7 ng/ml.

**Conclusion:**

Testosterone deficiency could be used as an independent predictor of symptom severity in HFpEF, and it aggravates systemic inflammation and endothelial dysfunction in these patients.

## Background

Heart failure with preserved Ejection Fraction (HFpEF) is challenging as patients usually have normal LV size and ejection fraction with increased ventricular stiffness and wall hypertrophy with increased fibrosis and/or change in left atrial size. In this heart failure phenotype, there is a complex interaction of several risk factors that cause organ dysfunction and clinical symptoms [1–3].

In different studies, the population suffering from heart failure worldwide was estimated to be more than 64 million subjects [4]. HFpEF prevalence was variable in different clinical studies depending on the definition and diagnostic criteria of the study. In different studies and registries of HFpEF, the incidence was between 19 and 55% of all patients with heart failure [4, 5].

In addition to changes in cardiac structure and function, some data suggest that endothelial and vascular dysfunction and increased inflammation may play a role in the pathophysiology of HFpEF [6]. Adhesion molecules, like intercellular adhesion molecule-1 (ICAM-1) and vascular cell adhesion molecule-1 (VCAM-1), are present in endothelial cells and participate in the process of dysfunction and inflammation [7].

There is evidence that Testosterone deficiency results in endothelial dysfunction by affecting the nitric oxide /cyclic guanosine monophosphate pathway, resulting in both erectile and vascular dysfunction [8].

The male gender is well-known as a risk factor for most cardiovascular diseases. Some studies showed that testosterone levels in men decrease gradually with aging, leading to a dramatic increase in the incidence of cardiovascular diseases [9]. The mechanism of abnormalities in cardiovascular performance with aging is not entirely understood. However, there is some evidence that androgen deficiency in men may aggravate the effect of traditional cardiovascular risk factors [10].

This work aimed to study the association between testosterone deficiency and endothelial dysfunction, increased inflammatory markers, and symptom severity in patients with HfpEF, opening the way for a new target in treating such complex syndrome.

## Methods

### Study population

This prospective cohort cross-sectional observational study was conducted on 120 male patients with the clinical syndrome of HFpEF patients. They were coming for follow-up in the outpatient clinic unit in the cardiology department between December 2020 and December 2021. The following criteria made the diagnosis of HEpEF; patients Ejection Fraction (EF) = 50% or more with 1- symptoms and signs of heart failure (dyspnea, orthopnea, lung crepitation, congested neck veins, lower limb edema), 2- the presence of any structural or functional cardiac abnormalities (Left ventricle mass index >_115 g/m2, E/e’ ratio at rest > 9, Left atrium volume index > 34 mL/m2) 3- BNP level > 35 pg/mL [11, 12].

**Fig. 1:**
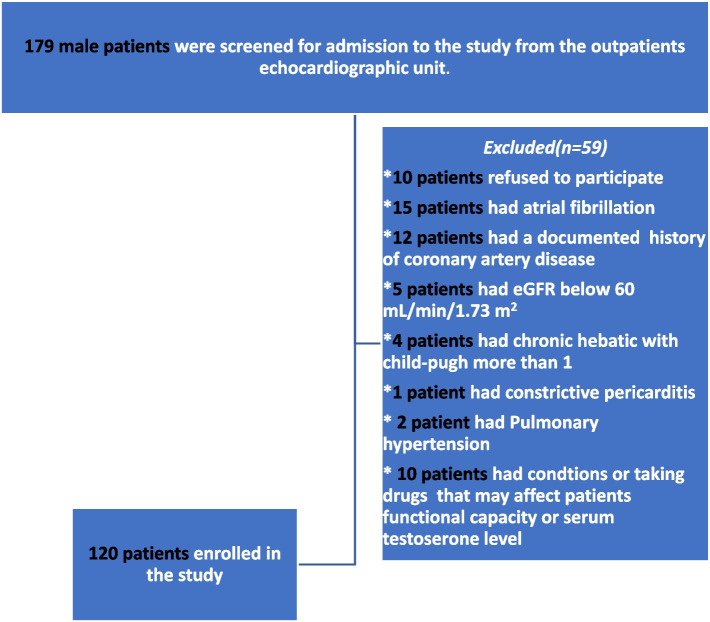
(patients flow Chart). eGFR: Estimated Glomerular Filteration Rate

Patients with EF less than 50%, uncontrolled blood pressure (≥ 180/110 mmHg), atrial fibrillation or any cardiac arrhythmias, recent admission with acute heart failure over the last 4 weeks, with documented ischemic heart disease, left bundle branch block on ECG, patients with severe heart failure symptoms New York Heart Association (NYHA) function class IV), pericardial disease, pulmonary hypertension, diabetes mellitus, or any endocrine conditions that may affect serum testosterone (e.g. thyroid dysfunction, Cushing’s syndrome), prescribed androgenic steroids, glucocorticoid, thyroid hormone, antithyroid drugs and/or medications that could affect serum testosterone levels (e.g., cimetidine, phenytoin, spironolactone), chronic liver disease, chronic chest disease, chronic kidney disease and/or anaemia were excluded. (a flow chart of the patients included is shown in Fig. 1).

All patients signed informed consent of participation, and the study protocol was approved by the local research committee and was according to the Helsinki ethical principles for medical research.

### Clinical evaluation

Patients were subjected to full history taking, including drug history and assessment of the severity of symptoms of heart failure using NYHA function class patients were asked to report their symptoms over the last month before enrollment in the study. NYHA I: no activity limitation; ordinary physical activity does not cause symptoms; NYHA II: slight activity limitation; ordinary physical activity causes symptoms; NYHA III: marked activity limitation; less than ordinary activity causes symptoms, NYHA IV: unable to carry any activity; symptoms at rest) [13] and calculation of body mass index (BMI) (body weight (kg) / height (m) squared) [14].

### Echocardiographic evaluation

Experienced echocardiographers performed transthoracic echocardiography, and images were stored and evaluated offline by two cardiologists not aware of the clinical data of the patients; to reduce interobserver variability. GE VIVID 9 machine was used for image acquisition.

Left ventricular ejection fraction (LVEF) was calculated using Simpson’s method in a 4-chamber view. Trans-mitral Left Ventricle (LV) filling velocities were evaluated using pulsed Doppler echocardiography. The peak early-diastolic flow velocity (E) and late-diastolic velocity (A) were presented as E/A ratio. Annular e’ velocity (septal e’ or lateral e’), average E/e′ ratio, maximum Left atrium (LA) volume index, and minimum LA volume index were evaluated. LA volumes were indexed to the body surface area. Left atrial ejection fraction (LA EF) % was calculated using the following equation (LAEF): (Maximum left atrium volume index (Max-LAVI) - Minimum left atrium volume index (Min-LAVI)/Max-LAVI × 100. Volumes were measured using the biplane-modified Simpson’s method. The.

two- dimensions (2D) volume of the LA from the apical view during a short breath-hold and 2D LA images from apical four- and two-chamber views were evaluated [15].

### Biochemical evaluation

The laboratory assessment of patients’ included 8-hour fasting blood glucose (FBG) by oxidase method using an assay kit (Biodiagnostic, Egypt), fasting insulin (FI), and the Homeostatic Model Assessment of Insulin Resistance (HOMA-IR). Insulin Resistance was diagnosed if the HOMA-IR was equal to or greater than 2.7 using the formula: HOMA-IR = fasting insulin in mIU/L x fasting glucose in mg/dL/405 [16].

Lipid profile, including total cholesterol (TC), triglycerides (TG), and high-density lipoprotein cholesterol (HDL), was measured by colorimetric methods using assay kits (Biodiagnostic, Egypt). Low-density lipoprotein (LDL) was calculated according to Friedewald et al.’s formula LDL = (TC) - (HDL) - (TG/5) [17].

Further, patients’ overnight fasting morning (before 11 am) total testosterone levels were assessed by a solid-phase enzyme immunoassay for the quantitative determination of testosterone in human serum ELISA Kit (ab174569), Abcam (Cambridge, UK) [18]. Testosterone deficiency was diagnosed if the total testosterone level was below 3.0 ng/ml [19]. Moreover, the B-natriuretic peptide (BNP) level was assessed by the BNP Human ELISA Kit (ab193694) Abcam (Cambridge, UK) provides for the Quantitative measurement of Natriuretic Peptides B in Serum [20] to evaluate heart failure state.

ICAM-1 was estimated using an ICAM-1 human ELISA kit (My Bio Source, Inc., CA, USA, (#MBS7600333).

P-Selectin was estimated using a human P-Selectin ELISA Kit (My Bio Source, Inc., CA, USA, (#MBS176001) according to the manufacturer’s instructions. Nitric oxide (NO) level was measured using Colorimetric Nitric Oxide assay kit (Mybiosource, USA) according to the manufacturer’s instructions.

### Statistical analysis

Statistical analysis was performed using the statistical package for the social sciences (SPSS, IBM Company USA) version 21. Data are presented as the mean ± SD value for continuous variables and as numbers and frequency percentages for categorical variables as descriptive analysis. All continuous variables were passed through the standard test for normality (Kolmogorov–Smirnov test). The analytical analysis also was done for comparison between categorical variables by using the X2 -test. A student t-test was used to compare two groups for normally distributed quantitative variables. Comparisons of continuous variables between groups were made using one-way ANOVA followed by the Scheffe post hoc test. Univariate and multivariable logistic regression analyses were performed to detect potential independent predictors of severe symptoms (NYHA III). Receiving operator characteristics (ROC) curve is used to detect optimal cutoff values for testosterone levels associated with severe symptoms (NYHA III). The level of significance was accepted if the *P*-value was less than 0.05.

## Results

### Comparison of baseline characteristics and laboratory findings in different NYHA classes

The baseline characteristic of subjects according to NYHA classes is given in Table 1. A total of 120 adult men with HFpEF were included in the study. According to the severity of symptoms (NYHA function class), the study population was divided into three groups 28 (23.33%) subjects with NYHA I, 56 (46.67%) subjects with NYHA II, and 36 (30%) subjects with NYHA III. Age was found to be significantly higher in subjects with NYHA III. Age in NYHA I, NYHA II and NYHA III groups were (50.29 ± 5.08), (52.73 ± 8.07) and (55.19 ± 5.40) years, respectively (*P =* 0.017). Moreover, diuretics use was significantly higher in the NYHA III group; 4 (14.33%), 12 (21.4%) and 16 (44.4%), respectively (*P =* 0.012).

**Table 1:** Comparision of clinical, demographic characteristics and laboratory findings between groups

	NYHA I (***n =*** 28)	NYHA II (***n =*** 56)	NYHA III (***n =*** 36)	F/X^**c**^	P	P1	P2	P3
**Age (Year)**	50.29 ± 5.08	52.73 ± 8.07	55.19 ± 5.40^c^	4.223	0.017^a^	0.119	0.005^a^	0.089
**BMI**	29.39 ± 3.75	29.91 ± 3.56	29.16 ± 2.99	0.577	0.563	0.513	0.786	0.305
**HTN history (%)**	9 (32.1%)	25 (44.6%)	20 (55.6%)	3.494	0.174	0.271	0.062	0.307
**SBP (mmHg)**	121.96 ± 11.49	124.73 ± 11.38	128.61 ± 11.63	2.747	0.098	0.300	0.051	0.116
**DBP (mmHg)**	82.14 ± 8.97	83.21 ± 7.89	85.42 ± 10.65	1.141	0.323	0.610	0.153	0.256
**FBG (mg/dl)**	89.32 ± 12.78	91.82 ± 11.61	94.83 ± 10.03	1.865	0.160	0.348	0.059	0.221
**FI (uIU/ml)**	9.90 ± 3.17	11.13 ± 4.21	12.24 ± 3.56	2.992	0.094	0.164	0.052	0.175
**HOMA-IR**	2.22 ± 0.88	2.51 ± 0.98	2.86 ± 0.95^c^	3.735	0.027^a^	0.179	0.008^a^	0.087
**TC (mg/dl)**	199.14 ± 48.30	210.23 ± 44.43	213.64 ± 38.81	0.930	0.397	0.276	0.191	0.716
**LDL (mg/dl)**	126.93 ± 40.11	129.56 ± 39.47	136.89 ± 34.26	0.628	0.536	0.766	0.302	0.370
**HDL (mg/dl)**	46.18 ± 7.15	44.71 ± 8.02	43.39 ± 7.49	1.045	0.355	0.411	0.151	0.420
**TGs (mg/dl)**	143.21 ± 34.38	148.73 ± 47.61	152.06 ± 29.47	0.388	0.679	0.552	0.382	0.698
**Hemoglobin (g/dl)**	11.70 ± 1.10	11.66 ± 1.00	11.53 ± 0.83	0.316	0.730	0.874	0.477	0.505
**BNP (Pg/ml)**	51.82 ± 9.54	58.34 ± 12.14^b^	70.36 ± 13.86^c,d^	19.870	0.001^a^	0.022^a^	0.001^a^	0.001^a^
**Testosterone (ng/ml)**	3.79 ± 1.54	2.96 ± 0.75^b^	2.49 ± 0.79^c,d^	13.408	0.001^a^	0.001^a^	0.001^a^	0.031^a^
**Testosterone deficiency (%)**	5 (17.9%)	9 (16.1%)	16 (44.4%)^c,d^	18.798	0.002^a^	0.001^a^	0.836	0.002^a^
**LAEF (%)**	51.21 ± 9.63	48.71 ± 8.48	44.28 ± 6.06^c,d^	6.169	0.003^a^	0.187	0.001^a^	0.012^a^
**ICAM-1 (ng/ml)**	372.25 ± 76.99	350.30 ± 75.91	429.75 ± 72.68^c,d^	12.382	0.001^a^	0.210	0.003^a^	0.001^a^
**P- selectin (ng/ml)**	250.32 ± 94.32	315.55 ± 71.80^b^	321.33 ± 95.45^c^	6.840	0.002^a^	0.001^a^	0.001^a^	0.750
**NO (μM)**	14.94 ± 1.15	13.94 ± 0.79^b^	13.75 ± 0.75^c^	16.544	0.001^a^	0.001^a^	0.001^a^	0.307
**ACEIs/ARBs (%)**	10 (35.7%)	22 (39.3%)	12 (33.3%)	0.349	0.840	0.751	0.842	0.564
**CCB (%)**	5 (17.9%)	12 (21.4%)	7 (19.4%)	0.159	0.924	0.701	0.872	0.819
**BB (%)**	4 (14.3%)	16 (28.6%)	12 (33.3%)	3.117	0.210	0.147	0.081	0.628
**Diuretics (%)**	4 (14.3%)	12 (21.4%)	16 (44.4%)^c,d^	8.779	0.012^a^	0.432	0.010^a^	0.019^a^

^a^**;** Statistical Significant Difference between all groups

^b^; (P1) Statistical Significant Difference in comparison to NYHA I & II

^c^; (P2) Statistical Significant Difference in comparison to NYHA I & III

^d^; (P3) Statistical Significant Difference in comparison to NYHA II & III

*BMI* Body Mass Index, *SBP* Systolic Blood Pressure, *DBP* Diastolic Blood Pressure

*HOMA-IR* Homeostatic Model Assessment of Insulin Resistance, *FI* Fasting Insulin

*FBG* Fasting Blood Glucose, *BNP* Brain Natriuretic Peptide, *LAEF* Left atrium Ejection Fraction

*ARBs* Angiotensin Receptor Blockers, *CCB* Calcium Channel Blockers, *BB* Beta Blocker

*NYHA* New York Heart Association

The cardio-metabolic profile patients in (NYHA III) had the worst profiles. The comparison of classes (I, II and III) showed that HOMA-IR were (2.22 ± 0.88), (2.51 ± 0.98) and (2.86 ± 0.95), respectively (*P =* 0.027). BNP were (51.82 ± 9.54), (58.34 ± 12.14), and (70.36 ± 13.86) Pg/ml, respectively (*P =* 0.001). LAEF were (51.21 ± 9.63%), (48.71 ± 8.48%) and (44.28 ± 6.062,3%), respectively (P = 0.003). Testosterone levels were (3.79 ± 1.54), (2.96 ± 0.75) and (2.49 ± 0.79) ng/ml, respectively (*P =* 0.001). A total of 30 cases had testosterone deficiency; their distribution in different groups was (17.9%), (16.1%) and (44.4%), respectively (*P =* 0.002).

Moreover, regarding endothelial function and inflammatory profiles, the comparison of classes (I, II and III) showed that ICAM-1 were (372.25 ± 76.99), (350.30 ± 75.91) and (429.75 ± 72.68) ng/ml, respectively (*P* = 0.001). P-selectin was (250.32 ± 94.32), (315.55 ± 71.80) and (321.33 ± 95.45) ng/ml, respectively (*P =* 0.002). NO was (14.94 ± 1.15), (13.94 ± 0.79) and (13.75 ± 0.75) μM, respectively (*P =* 0.001).

### Comparison of the characteristics of patients with & without testosterone deficiency (Table 2)

A total of 30 patients (25%) of the studied population had testosterone deficiency. In this group of patients (17.6%), (16.1%) and (44.4%) of them were in NYHA I, NYHA II and NYHA III classes, respectively (*P* = 0.002). The diuretic use was significantly higher in the testosterone deficiency group; (50%) versus (18.9%) in patients without testosterone deficiency (*P* = 0.007); this may reflect the impact of more severe symptoms in patients with a deficiency which leads to more prescription of diuretics to relieve symptoms.

**Table 2:** comparison between patients with and without testosterone deficiency

	No Testosterone Deficiency (***n =*** 90)	Testosterone deficiency (***n =*** 30)	t / X^**2**^	P
**Age (Year)**	51.79 ± 7.48	53.35 ± 6.66	1.133	0.259
**HTN history (%)**	35 (40.7%)	16 (55.9%)	2.720	0.132
**BMI**	29.26 ± 3.57	30.32 ± 2.97	1.535	0.128
**SBP (mmHg)**	121.40 ± 10.45	135.00 ± 8.44	6.764	0.001^a^
**DBP (mmHg)**	81.80 ± 8.08	88.24 ± 9.84	3.690	0.001^a^
**FBG (mg/dl)**	91.08 ± 11.17	94.82 ± 12.17	1.612	0.110
**FI (uIU/ml)**	10.36 ± 3.73	13.25 ± 3.44	3.907	0.001^a^
**HOMA-IR**	2.33 ± 0.92	3.09 ± 0.87	4.139	0.001^a^
**TC (mg/dl)**	201.65 ± 43.49	226.41 ± 39.75	2.877	0.005^a^
**LDL (mg/dl)**	123.68 ± 35.43	150.03 ± 38.31	3.587	0.001^a^
**HDL (mg/dl)**	44.17 ± 7.49	45.88 ± 8.09	1.100	0.274
**TGs (mg/dl)**	144.15 ± 40.28	159.29 ± 36.80	1.900	0.060
**Hemoglobin (g/dl)**	11.55 ± 0.90	11.84 ± 1.11	1.461	0.147
**BNP (Pg/ml))**	56.10 ± 12.52	71.35 ± 11.22	6.183	0.001^a^
**LAEF (%)**	50.01 ± 8.62	42.79 ± 5.43	4.533	0.001^a^
**ICAM-1 (ng/ml)**	356.35 ± 78.64	437.21 ± 59.54	5.408	0.001^a^
**P- selectin (ng/ml)**	281.49 ± 84.77	354.12 ± 78.19	4.320	0.001^a^
**NO (μM)**	14.42 ± 0.94	13.34 ± 0.57	6.170	0.001^a^
**ACEIs/ARBs (%)**	35 (40.7%)	9 (26.5%)	2.124	0.145
**CCB (%)**	16 (18.6%)	8 (23.5%)	0.369	0.543
BB (%)	21 (24.4%)	11 (32.4%)	0.784	0.376
**Diuretics (%)**	17 (19.8%)	15 (50%)	7.388	0.007^a^
**NYHA class**				
**NYHA I (%)**	23 (26.7%)	5 (14.7%)	18.798	0.001^a^
**NYHA II (%)**	47 (54.7%)	9 (26.5%)		
**NYHA III (%)**	16 (18.6%)	20 (58.8%)		

*BMI* Body Mass Index, *SBP* Systolic Blood Pressure, *DBP* Diastolic Blood Pressure

*HOMA-IR* Homeostatic Model Assessment of Insulin Resistance, *FI* Fasting Insulin

*FBG* Fasting Blood Glucose, *BNP* Brain Natriuretic Peptide, *LAEF* Left atrium Ejection Fraction

*ARBs* Angiotensin Receptor Blockers, *CCB* Calcium Channel Blockers, *BB* Beta Blocker

*NYHA* New York Heart Association ^a^; Statistical Significant

Regarding cardio-metabolic profiles, the testosterone deficiency group showed significantly higher systolic and diastolic blood pressure (*P* = 0.001), Fasting insulin (*P =* 0.001), HOMA-IR (*P =* 0.001), total cholesterol (*P* = 0.005), LDL (*P =* 0.001), BNP (*P =* 0.001) and LAEF (*P =* 0.001).

Moreover, regarding endothelial function and inflammatory profiles, the testosterone deficiency group showed significantly higher ICAM-1 (*P =* 0.001) and P-selectin (*P =* 0.001) and significantly lower NO (*p* = 0.001).

### Multivariate and ROC curve analysis

We performed univariate and multivariate analyses to find our patients’ independent predictors of NYHA class III (Table 3). Age, BNP and testosterone levels be used as independent predictors of NYHA class III with (*P* = 0.034, 0.007 and 0.008), respectively.

**Table 3:** Univariate and multivariate analysis of predictors of severe symptoms (NYHA III)

	Univariate		Multivariate	
	OR (95% CI)	***P*** value	OR (95% CI)	***P*** value
**Age (Years)**	1.854 (1.147–2.851)	0.011^a^	1.305 (1.085–2.931)	0.034^a^
**HOMA-IR**	1.352 (0.854–2.415)	0.093		
**BNP (Pg/ml)**	2.851 (1.327–4.861)	0.001^a^	2.052 (1.754–4.732)	0.007^a^
**Testosterone (ng/ml)**	0.594 (0.317–0.761)	0.001^a^	0.634 (0.471–0.864)	0.008^a^
**LAEF %**	0.417 (0.234–0.607)	0.013^a^	0.854 (0.596–1.764)	0.108

*HOMA-IR* Homeostatic Model Assessment of Insulin Resistance

*BNP* Brain Natriuretic Peptide, *LAEF* Left atrium Ejection Fraction

^a^**;** Statistical Significant

**Fig. 2:**
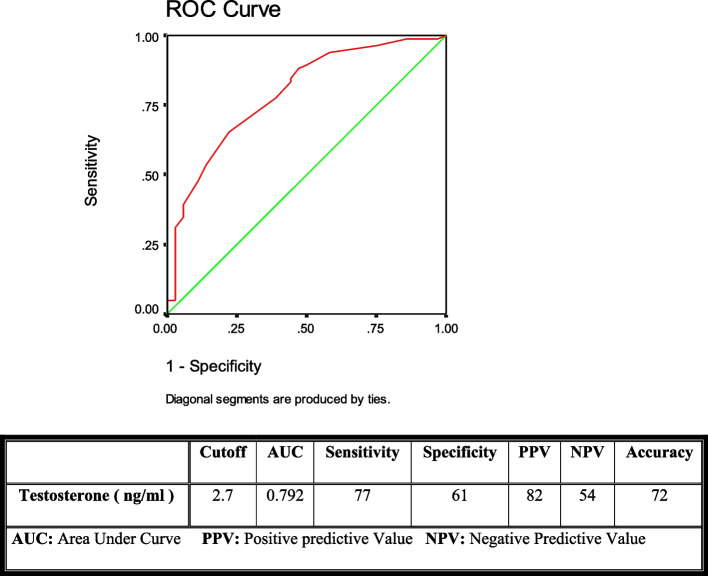
ROC Curve for testosterone best cutoff value that predicts severe symptoms (NYHA class III)

Finally, the ROC curve was done to detect the optimal cutoff value of testosterone level that can predict severe symptoms (NYHA III), and we found it at 2.7 ng/ml with the area under the curve (0.792), sensitivity (78%) and Specificity (61%) (Fig. 2).

## Discussion

Heart failure with preserved ejection fraction pathophysiology is poorly understood. The ventricle’s ability to relax and fill during diastole is affected by multiple factors, including plasma volume, structural characteristics of the LV wall, active energy-driven processes involved in LV relaxation, atrial contraction, and the integrity of the mitral valve [21]. Nonetheless, HFpEF is relatively common and presents significant challenges in diagnosis and treatment [22]. Our study extends previous knowledge by demonstrating potential predictors of symptoms in HFpEF patients with its pathophysiological aspects.

HFpEF is associated with a state of inflammation in the endothelium of microvasculature that leads to endothelial dysfunction and reduced NO production and function with increased oxidative stress; this was proved by Franssen et al. [23].

In this study, we found that patients with severe symptoms were elderly, had higher systolic and diastolic blood pressure at enrollment, and had a higher incidence of testosterone deficiency which could be used as an independent predictor of the severity of symptoms also; they had increased markers of systemic inflammation, endothelial dysfunction and insulin resistance.

**Fig. 3:**
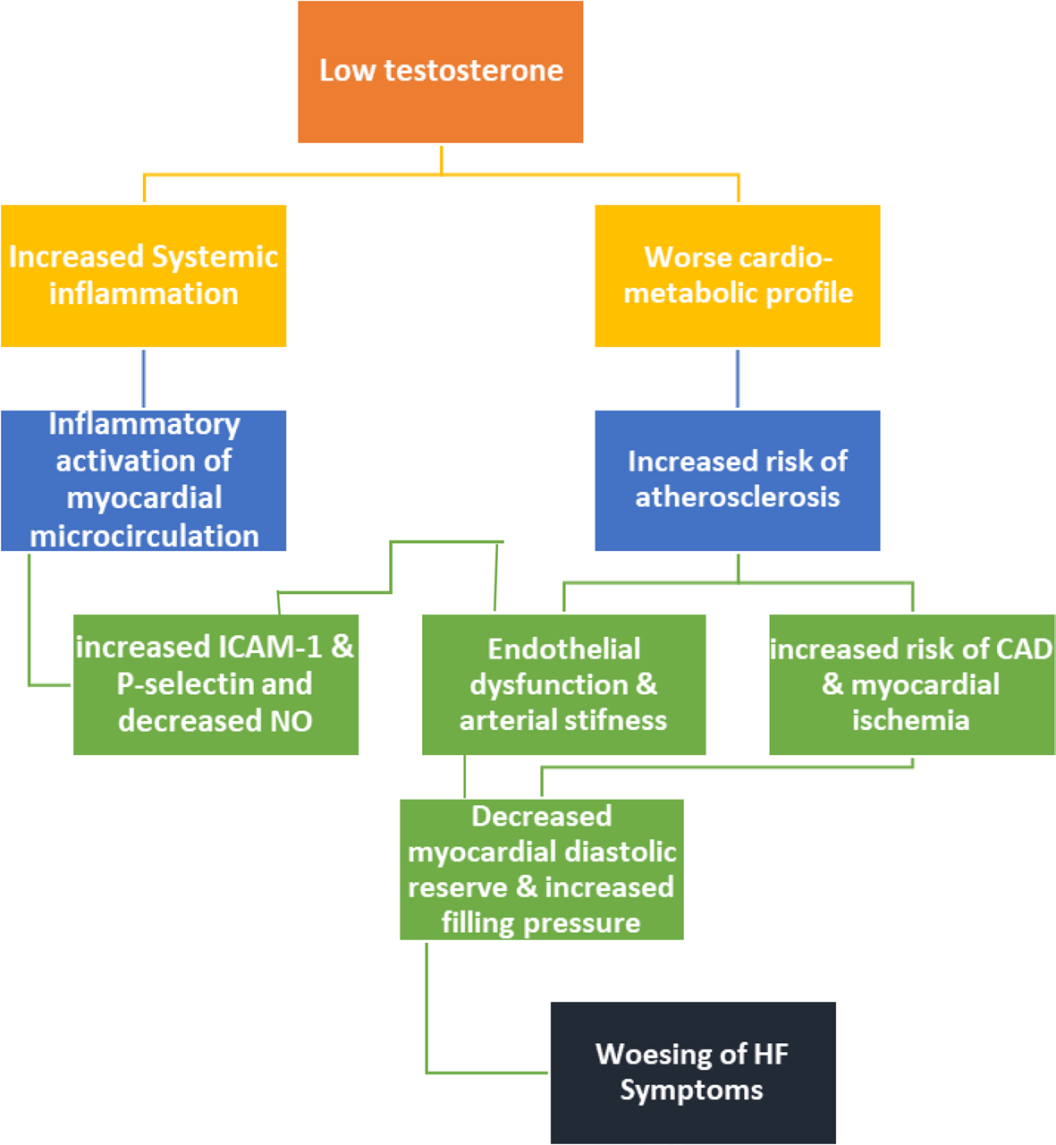
Illustration of the possible mechanism that links testosterone deficiency to the worsening symptoms of HFpEF

On the other hand, testosterone deficiency patients had higher markers of heart failure, worse metabolic profiles, more elevated inflammatory markers, and low NO levels. Our suggested mechanism linking the worsening of symptoms of heart failure to the co-existing testosterone deficiency was illustrated in (Fig. 3).

Dermitas et al. found that testosterone deficiency in the aged rate was associated with an increased level of tumor necrosis factor α (TNF-α), C-reactive protein (CRP), and intercellular adhesion molecule (ICAM-1) levels [24]. and We found in our cohort that patients with testosterone deficiency had significantly higher levels of and (ICAM-1). (ICAM-1) is a cell surface glycoprotein typically expressed in endothelial and immune system cells. The increase in its expression is associated with some immune and inflammatory responses like increased leukocyte recruitment [25].

Albar Z et al. concluded that the increase in inflammatory markers is a risk factor for HFpEF [26]. Furthermore, Hage et al. [27] found in their study that inflammatory markers in HFpEF patients can predict symptom severity and prognosis.

We found that NO level was significantly lower in HFpEF who had testosterone deficiency compared to those with a normal level.

Previous studies showed that the myocardium in HFpEF demonstrated upregulation of p-selectin and intercellular adhesion molecule-1 expression levels and uncoupling of endothelial NO synthase associated with reduced myocardial nitrite/nitrate concentration, cGMP content, and Protein Kinase-G activity [23]. Furthermore, Sansone et al., in their meta-analysis, found that testosterone replacement therapy improved endothelial function and flow-mediated dilation [28]. Borlaug et al. [6] suggested that In patients with HEpEF, the exercise capacity (dyspnea and fatigue with submaximal exercise test) was negatively correlated to the degree of flow-mediated vasodilatation, which is a marker of NO availability and endothelial function. This matches our data, showing that patients with severe symptoms (NYHA III) had a higher level of P-selectin, ICAM-1 and a lower NO level.

Zhou et al. found that Metabolic syndrome and insulin resistance were associated with poor outcomes and increased incidence of hospitalization in HFpEF [29]. We found that patients with testosterone deficiency had a worse metabolic profile, which may play a role in worsening their symptoms. Additionally, testosterone also influences skeletal muscle strength and increases muscle mass [30]. Skeletal muscle is thought to be the body’s primary site for glucose uptake because it receives glucose and insulin via capillary recruitment, transendothelial diffusion, activation of insulin receptors, resulting in intracellular signalling, and translocation of the glucose transporter 4 (GLUT4) to the cell membrane and finally glucose uptake. Therefore, losing muscle mass associated with testosterone deficiency may worsen insulin resistance [31].

Insulin resistance which may be a consequence of testosterone deficiency, is strongly associated with the risk of HFpEF progression. Insulin resistance and associated systemic inflammatory state, including secretion of pro-inflammatory cytokines, ultimately predispose to myocyte remodelling and the development and progression of HFpEF [32].

The metabolic abnormality in our patients with testosterone deficiency also extended to the lipid profile. Patients who had deficiency showed higher total cholesterol, triglycerides, and LDL levels without significant differences in HDL levels. Grandyset al [33]. concluded that testosterone level negatively correlates to total cholesterol, triglycerides, and LDL and has a non-significant correlation to HDL. These data ultimately are matched with our results. These metabolic changes may increase the atherosclerosis burden and increase the incidence of myocardial ischemia and microvascular dysfunction, which leads to more aggravation of heart failure symptoms.

We found an association between the severity of symptoms and age. Many, if not all, of the pathophysiological elements of HFpEF are affected by cardiac ageing. Age-related changes in structure and function have been recognized as important contributing factors to HFpEF, including ventricular vascular stiffening, vascular dysfunction, reduced calcium control, diminished adrenergic reserve, and physical deconditioning [34].

In our study, there was a positive correlation between the severity of symptoms in HFpEF patients and BNP levels, and its level can independently predict symptom severity in those patients. The B-type natriuretic peptide is a 32-amino acid peptide mainly released by the heart in response to increased myocardial wall stress due to volume or pressure overload [35]. Increasing levels of BNP predict a worse prognosis in all heart failure phenotypes in a linear fashion [36].

In our study, NYHA class III showed lower LAEF than NYHA class I and NYHA class II groups. In addition, there was a negative correlation between the severity of symptoms and LAEF. This finding was comparable with what Khan et al. concluded in their meta-analysis [37].

Also, previous studies reported that left atrium ejection fraction was lower in HFpEF compared to age- and sex-matched healthy controls. Lower LAEF is linked to bad outcomes in HFpEF and is also an independent predictive marker in HFpEF. It was associated with LA volumes and plasma indicators of atrial stress/stretch [38].

## Conclusion

In HFpEF patients, testosterone deficiency is associated with worsening heart failure symptoms. The more severe symptoms could be attributed to testosterone deficiency’s increased systemic inflammation and endothelial dysfunction. Also, testosterone deficiency is associated with a worse cardio-metabolic profile which may play a role in the progression of symptoms. Therefore, hormonal replacement therapy for these patients could provide a novel target for treating patients with HFpEF. A further study on a larger number of patients is recommended to validate our results.

### Study limitations

The limitations of our study include the cross-section design and the relatively small number of patients. In addition, we excluded patients with atrial fibrillation. Also, we did not investigate the cause of the deficiency, and we did not measure serum estradiol levels, total urinary testosterone, and its metabolites or report any anatomical abnormalities of the genitals.

## Data Availability

Are available on request from the corresponding author.

## Abbreviations

HFpEF: Heart Failure with Preserved Ejection Fraction
ICAM-1: Intercellular Adhesion Molecule-1
VCAM-1: Vascular Cell Adhesion Molecule-1
NYHA: New York Heart Association
BMI: Mass Index
LVEF: Left Ventricular Ejection Fraction
LV: Left Ventricle
LA: Left Atrium
LAEF: Lef Atrial Ejection Fraction
Max-LAVI: Maximum Left Atrium Volume Index
Min-LAVI: Minimum Left Atrium Volume Index
2D: Two-Dimensions
FBG: Fasting Blood Glucose
FI: Fasting Insulin
HOMA-IR: Homeostatic Model Assessment of Insulin Resistance
TC: Total Cholesterol
TG: Triglycerides
HDL: High-Density Lipoprotein
LDL: Low-Density Lipoprotein
BNP: B-Natriuretic Peptide
NO: Nitric oxide
ROC: Receiving Operator Characteristics
